# The mediating role of transition shock between work readiness and workplace adaptability in newly graduated nurses: A cross-sectional study

**DOI:** 10.1097/MD.0000000000046268

**Published:** 2025-11-28

**Authors:** Xiang Yi, Guoqing Wang

**Affiliations:** a Department of Organ Transplantation, Renmin Hospital of Wuhan University, Wuhan, Hubei, China; b Department of Eastern Hospital Dialysis Center, Renmin Hospital of Wuhan University, Wuhan, Hubei, China.

**Keywords:** nurses, nursing education, nursing management, role transition, transition shock, work readiness, workplace adaptability

## Abstract

This study aimed to investigate the mediating role of transition shock (TS) in the relationship between work readiness and workplace adaptability among newly graduated nurses by constructing a structural equation model. In a cross-sectional survey, 208 newly graduated nurses were selected from 2 tertiary hospitals (Wuhan, China), and data were collected using demographic questionnaires, the work readiness scale for graduate nurses, nurses’ workplace adaptability scale, and TS scale of newly graduated nurses. A structural equation model was employed to investigate the mediating role of TS in the relationship between work readiness and workplace adaptability. Statistical analyses were conducted using IBM SPSS software, version 21.0 and SPSS AMOS 24.0 (IBM Corporation, Armonk). The statistical tests conducted in this study were 2-tailed, and a *P* value of <.05 was considered significant. Significant associations were found among work readiness, TS, and workplace adaptability (*P* < .01). In the mediation models, TS partially mediated the relationships between work readiness and workplace adaptability (indirect effect: 0.169, 95% confidence interval (CI): 0.081–0.300, *P* < .01; direct effect: 0.215, 95% CI: 0.051–0.376, *P* < .05; total effect: 0.384, 95% CI: 0.241–0.521, *P* < .01), and the mediating effect accounted for 44.01% (0.169/0.384 × 100%) of the total effect. This indirect effect could be considered moderate mediation. A significant relationship was found between work readiness, TS, and workplace adaptability among newly graduated nurses. Work readiness indirectly affected workplace adaptability through TS. These findings hold direct practical implications for clinical nursing management. The results demonstrated that TS was a critical mediator, suggesting that interventions designed to reduce it were likely to enhance new nurses’ workplace adaptability. Therefore, nursing managers and educators should prioritize developing structured support programs, such as enhanced mentorship programs, peer mentoring, and resilience training workshops, to buffer its negative effects. Concurrently, efforts to improve work readiness during nursing education remained essential. Implementing such programs could improve workplace adaptability, thereby stabilizing the nursing team and fostering sustainable development.

## 1. Introduction

Newly graduated nurses referred to those who had worked for 2 years after graduation.^[1]^ With the development of the global economy and medical careers, the social demand for nursing professionals is increasing.^[2]^ However, various pressures faced by nursing staff have led to increasing demission, which exacerbates the loss of nursing professionals and hinders their career development.^[3]^ The acute shortage of nurses is a global problem, with the current workforce struggling to meet the demands of this rapidly expanding medical profession.^[4]^ In this challenging situation, facilitating the adaptation of new nurses to clinical work is essential for transforming them into professional nurses and medical managers.

Workplace adaptability refers to an individual’s ability to adjust to the work environment and promote harmonious relationships.^[5]^ In the context of nursing, workplace adaptability can significantly impact the professional experience and work enthusiasm of nurses, thus affecting the quality of hospital care and the stability of the overall nursing team.^[6,7]^ Issues with workplace adaptability are notably pronounced among newly graduated nurses.^[8,9]^ The contributing factors include the lack of work experience, difficulties in adapting to clinical settings, inadequate skills in handling clinical emergencies, and critical phases of workplace adaptability and role transition.^[10]^

Transition shock (TS) could be understood as the feeling and experience of confusion, doubt, and disorientation in areas such as physical well-being, psychology, knowledge and skills, social culture, and career development. These feelings arise due to the shift in knowledge, responsibility, role, and relationship as newly graduated nurses transition from a student role to a nurse role.^[11,12]^ Relevant studies have found a significant negative correlation between TS and workplace adaptability among newly graduated nurses.^[13,14]^ Prolonged high levels of TS not only cause negative physical and psychological effects on newly graduated nurses but also lead to a decrease in enthusiasm for learning professional knowledge and skills. This can lead to professional aversion and turnover intention, severely impacting their career development.^[15]^

Work readiness is the extent to which an individual is equipped with the personal traits and attitudes necessary for achieving success in the workplace.^[16]^ Higher levels of work readiness may improve workplace adaptability outcomes.^[17]^ However, the current state of work readiness among newly graduated nurses is concerning. A survey^[18]^ on the ability and practical readiness of newly graduated nurses revealed that only 23% of nurses had reached the entry-level standard. Additionally, hospital administrators and clinical nursing educators had expressed concerns about the work readiness in newly graduated nurses.^[19,20]^

Based on the literature review, this research proposes the weakness of the previous studies. TS, work readiness, and workplace adaptability are closely related,^[13,14,17,21,22]^ but the dynamics of how these factors influence each other have not yet been explored, given the lack of empirical studies among newly graduated nurses. Therefore, this study aims to explore and analyze the potential mechanisms underlying workplace adaptability by examining the relationships between TS, work readiness, and workplace adaptability. The findings from this study will contribute to future research on improving workplace adaptability in newly graduated nurses and provide valuable insights for nursing managers to design effective nurse support programs. Implementing such programs could improve workplace adaptability, thereby stabilizing the nursing team and fostering sustainable development.

This study used the TS theory as the theoretical framework.^[11]^ According to this theory, the professional role transition for newly graduated nurses is a process of adjustment that encompasses developmental, intellectual, sociocultural, and physical aspects, all driven and mediated by shifts in roles, responsibilities, relationships, and levels of knowledge in their personal and professional lives. This theory suggests that educational institutions and industry employers should focus on providing senior nursing students with comprehensive preparatory education about role transition; facilitating clinical placements that more effectively prepare graduates for the dynamic, highly intense, and conflict-laden environment of professional practice; and expanding workplace orientation programs to ensure a balanced integration of theoretical knowledge and practical clinical skills. According to the TS theory, work readiness plays an important role in TS and workplace adaptation. Moreover, TS plays a significant role in influencing workplace adaptation.

**Figure 1.**
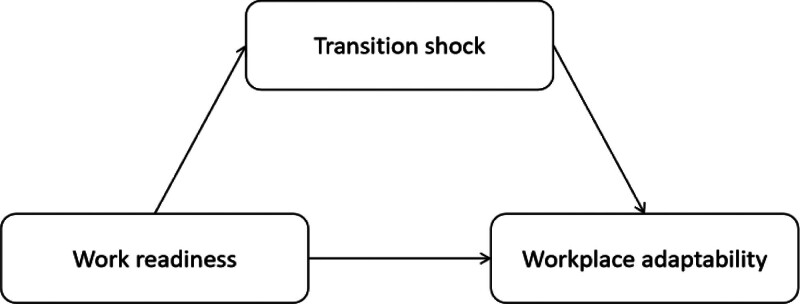
Concept diagram of the mediating effect model.

Based on the literature review and theoretical framework, we hypothesized that TS mediates the relationship between work readiness and workplace adaptability. A structural equation modeling will be used to evaluate the proposed hypotheses. Structural equation modeling is a statistical methodology that takes a confirmatory (i.e., hypothesis-testing) approach to the analysis of a structural theory bearing on some phenomenon.^[23]^ Accordingly, a concept diagram for the mediating effect model was developed (Fig. 1).

## 2. Materials and methods

### 2.1. Design

This cross-sectional study was conducted in Wuhan, China, from May to August 2022. This study was carried out in Wuhan owing to its advanced medical infrastructure, numerous hospitals, a large population of medical students, and the representativeness of its newly graduated nurses.

### 2.2. Participants and settings

Convenience sampling was also performed, and newly graduated nurses from 2 tertiary hospitals (Wuhan, China) were invited to participate in this study. The participants completed an online questionnaires. Registered nurses who had obtained practicing certificates and had worked for 2 years or less, newly graduated nurses who had completed prework training and engaged in clinical nursing work, and newly graduated nurses who were willing to participate in the survey and signed the informed consent form were included in the study. Newly graduated nurses who had been off duty for more than 1 month due to various reasons were excluded. This criterion was implemented because such an extended absence from the clinical environment could significantly alter the natural trajectory of TS and the process of adapting to the workplace, thereby confounding the assessment of the relationships between work readiness, TS, and workplace adaptability.

To estimate the required sample size, quantitative methods commonly used in nursing research were applied.^[24]^ This method considers that the sample size should be proportionate to the number of independent variables, typically requiring 5 to 10 times the number of variables. The general data questionnaire included 7 items, which were subdivided into 5 dimensions of work readiness, 3 dimensions of workplace adaptability, and 4 dimensions of TS. To account for a potential 20% rate of invalid responses, the desired sample size was 114 to 228 participants. Thus, the inclusion of 208 participants exceeded this requirement, thereby satisfying the estimation requirements.

Before participating in the study, the participants were informed about the purpose of the research, its significance, and data security measures. In addition, participation was voluntary and anonymous; the participants were informed of their rights and responsibilities and had the right to withdraw from the study at any time. This study was approved by the ethics committee of Renmin Hospital of Wuhan University (No. WDRY2023-K053). In addition, written informed consent was obtained from all participants.

### 2.3. Instruments

#### 2.3.1. Sociodemographic characteristics

The demographic data obtained from newly graduated nurses included sex, age, educational level, having 1 child, degree of family support, hospital practice before entry, and location of family.

#### 2.3.2. Work readiness scale for graduate nurses

Walker et al developed a work readiness scale for graduate nurses^[17]^ to assess their level of work readiness. The Chinese version of the scale was translated by Li.^[25]^ It consists of 37 items across 5 dimensions. All items were measured on a 10-point Likert scale (1 = strongly disagree to 10 = strongly agree), with higher scores indicating a higher level of work readiness. The total score ranges from 37 to 370 points. In this study, the Cronbach’s α coefficient for this scale was 0.960, suggesting high reliability.

#### 2.3.3. Nurses’ workplace adaptability scale

The nurses’ workplace adaptability scale, developed by Fujjmoto et al,^[26]^ was used to evaluate the workplace adaptability level of newly graduated nurses. The Chinese version of the scale was translated and revised by Liu et al.^[27]^ This questionnaire comprised 17 items measured on a 5-point Likert scale, ranging from 1 (did not apply to me at all) to 5 (applied to me very much or most of the time). Higher scores indicated a higher level of workplace adaptability, with the total score ranging from 17 to 85 points. In this study, the Cronbach’s α coefficient for this scale was 0.889.

#### 2.3.4. Transition shock of newly graduated nurses scale

The TS of the newly graduated nurses scale, developed by Xue et al^[28]^ in 2015, was adopted in this study. It consists of 27 items across 4 dimensions. All items were measured on a 5-point Likert scale (1 = did not apply to me at all to 5 = applied to me very much or most of the time), with higher scores indicating a higher level of TS. The mean scores for each dimension were categorized as follows: <2.17 for low level, 2.17 to 3.83 for medium level, and >3.83 for high level.^[29]^ In this study, Cronbach’s α coefficient for this scale was 0.971.

### 2.4. Data collection

After obtaining consent from the nursing heads of various hospitals, the researchers sent the questionnaires online to newly graduated nurses. The study’s purpose, informed consent details, and instructions for completion were provided on the front page of the questionnaire. Newly graduated nurses completed the questionnaire either independently or with guidance from a researcher. The researchers received professional training, and each phone number was permitted to submit responses only once. Questionnaires completed within <180 s or exhibiting obvious response patterns were considered invalid. The questionnaire was checked and verified, and data were entered by 2 researchers to ensure accuracy.

### 2.5. Statistical analysis

IBM SPSS 21.0 and AMOS24.0 were used to analyze the data. The data satisfied the assumption of normality. Descriptive data included frequencies, percentages, mean values, and standard deviations. Pearson’s correlation coefficients were used to analyze the associations between variables. The Harman single-factor common method was employed to assess common method bias. AMOS24.0 was used for confirmatory factor analysis and structural equation model construction. Model fit was evaluated using a fixed index, and the model was adjusted by modifying the index. Bootstrapping was used to test the mediating effects. A *P* value of <.05 was considered significant.

## 3. Results

### 3.1. Participant characteristics

A total of 232 questionnaires were distributed to newly graduated nurses. We received 221 submitted responses, yielding an initial response rate of 95.26%. After excluding 13 invalid questionnaires, 208 valid responses were retained for data analysis, resulting in an effective response rate of 94.12%. The participants were aged 20 to 28 years, with an average age of 21.93 ± 1.16 years. Additional general information is provided in Table 1.

**Table 1:** Characteristics of the participants (N = 208).

Variable	N(%)	Mean ± SD
Sex		
Female	178(85.6)	
Male	30(14.4)	
Age (yr)	–	21.93 ± 1.16
Education		
Below bachelor’s degree	34(16.3)	
Bachelor’s degree or above	174(83.7)	
One child		
Yes	84(40.4)	
No	124(59.6)	
Degree of family support		
Hardly ever	2(1.0)	
General	86(41.3)	
Superior	120(57.7)	
Practice hospital before entry		
Secondary hospital	10(4.8)	
Tertiary hospital	198(95.2)	
Location of family		
Wuhan local	178(85.6)	
Outside Wuhan	30(14.4)	

SD = standard deviation.

### 3.2. Common method bias test

The Harman single-factor common method was used to assess common method bias. The results indicated the presence of 7 factors with eigenvalues of >1, with the first factor explaining 35.57% of the variance. This percentage was below the critical standard of 40%.^[30]^ The absence of a single dominant factor or an unusually high factor loading suggests that serious common method bias and variation were not present.

### 3.3. Work readiness, workplace adaptability, and transition shock scores of newly graduated nurses

The work readiness, TS, and workplace adaptability scores were 272.11 ± 45.70, 79.94 ± 19.33, and 60.98 ± 8.19, respectively. The details are listed in Table 2.

**Table 2:** Work readiness, workplace adaptability, and transition shock scores of newly graduated nurses (N = 208).

Variable	Number of items	Range of score	Score	Equalization of items
Work readiness	37	37–370	272.11 ± 45.70	7.35 ± 1.24
Work competence	8	8–80	55.51 ± 11.17	6.94 ± 1.40
Social intelligence	9	9–90	64.61 ± 12.59	7.18 ± 1.40
Career development	9	9–90	66.60 ± 12.86	7.40 ± 1.43
Organizational acumen	7	7–70	59.72 ± 10.44	8.53 ± 1.49
Personal work characteristics	4	4–40	25.67 ± 7.11	6.42 ± 1.78
Workplace adaptability	17	17–85	60.98 ± 8.19	3.59 ± 0.48
Work environment and atmosphere	11	11–55	41.31 ± 5.39	3.76 ± 0.49
Work autonomy	2	2–10	6.85 ± 1.33	3.43 ± 0.67
Relationship with superiors	4	4–20	12.83 ± 2.95	3.21 ± 0.74
Transition shock	27	27–135	79.94 ± 19.33	2.96 ± 0.72
Physical	6	6–30	19.30 ± 5.68	3.22 ± 0.95
Psychology	8	8–40	22.42 ± 6.07	2.80 ± 0.76
Knowledge and skills	5	5–25	16.12 ± 3.67	3.22 ± 0.73
Social culture and development	8	8–40	22.10 ± 6.46	2.76 ± 0.81

### 3.4. Correlation analysis of work readiness, workplace adaptability, and transition shock in newly graduated nurses

Pearson’s correlation analysis showed that work readiness was positively correlated with workplace adaptability (*r* = 0.404, *P* < .01), while person-organization fit was negatively correlated with work readiness and workplace adaptability (*r* = −0.472 and −0.368, *P* < .01 for all). The details are listed in Table 3.

**Table 3:** Correlation analysis of work readiness, workplace adaptability, and transition shock in newly graduated nurses (*r*).

Variable	1	2	3	4	5	6	7	8	9	10	11	12	13	14	15
1 Work readiness	1.000														
2 Work competence	0.884**	1.000													
3 Social intelligence	0.920**	0.808**	1.000												
4 Career development	0.948**	0.787**	0.858**	1.000											
5 Organizational acumen	0.781**	0.530**	0.620**	0.773**	1.000										
6 Personal work characteristics	0.547**	0.478**	0.411**	0.395**	0.223**	1.000									
7 Workplace adaptability	0.404**	0.405**	0.340**	0.404**	0.322**	0.152*	1.000								
8 Work environment and atmosphere	0.474**	0.419**	0.406**	0.474**	0.455**	0.142*	0.914**	1.000							
9 Work autonomy	0.360**	0.436**	0.300**	0.344**	0.171*	0.223**	0.785**	0.603**	1.000						
10 Relationship with superiors	0.093	0.163*	0.067	0.101	−0.015	0.063	0.750**	0.438**	0.625**	1.000					
11 Transition shock	−0.472**	−0.420**	−0.478**	−0.448**	−0.366**	−0.181**	−0.368**	−0.308**	−0.375**	−0.289**	1.000				
12 Physical	−0.418**	–0.355**	–0.418**	–0.384**	–0.345**	–0.188**	–0.378**	–0.339**	–0.341**	–0.273**	0.878**	1.000			
13 Psychology	–0.396**	–0.344**	–0.434**	–0.356**	–0.282**	–0.178**	–0.262**	–0.201**	–0.267**	–0.240**	0.899**	0.724**	1.000		
14 Knowledge and skills	–0.356**	–0.361**	–0.360**	–0.333**	–0.167*	–0.234**	–0.217**	–0.119	–0.344**	–0.229**	0.835†	0.608**	0.726**	1.000	
15 Social culture and development	–0.471**	–0.416**	–0.450**	–0.479**	–0.433**	–0.076	–0.398**	–0.365**	–0.375**	–0.269**	0.902**	0.724**	0.702**	0.714**	1.000

* *P* < .05.

** *P*<.01

### 3.5. Mediation analysis

**Figure 2.**
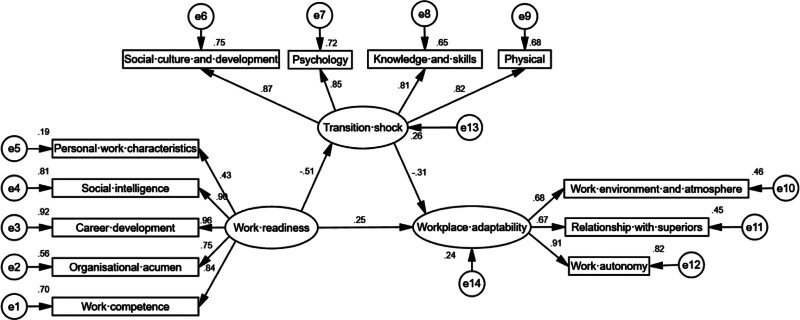
The finalized structural model (N = 208). Factor loadings are standardized.

A structural equation model was developed, with work readiness as the independent variable, workplace adaptability as the dependent variable, and TS as the intermediary variable. Maximum likelihood estimation was used for model fitting. The fix indices for the modified model were as follows: chi-square to degrees of freedom = 2.962 (<3), goodness of fit index = 0.905 (>0.9), normed fit index = 0.924 (>0.9), comparative fit index = 0.947 (>0.9), incremental fit index = 0.948 (>0.9), and root mean square error of approximation = 0.079 (<0.08), indicating a good model fit (Fig. 2). The bootstrap confidence interval estimation method was used for interval estimation, and 2000 samples were selected from the 208 original data by random sampling method. The 95% bootstrap confidence interval did not include 0, indicating that the TS partially mediated the relationship between work readiness and workplace adaptability. The mediating effect accounted for 44.01% (0.169/0.384 × 100%) of the total effect. The details are listed in Table 4.

**Table 4:** Bootstrapping results for the total effect, indirect effect, and direct effect of the meditational model.

Effect	Value of effect	95% CI		*P*	Effect proportion(%)
		Lower	Upper		
Indirect effect	0.169	0.081	0.300	.001	44.01
Direct effect	0.215	0.051	0.376	.011	55.99
Total effect	0.384	0.241	0.521	.001	100.00

CI = confidence interval.

## 4. Discussion

The results of this study showed that the workplace adaptability score of newly graduated nurses was 60.98 ± 8.19, aligning with the results of Liu et al’s study^[27]^ and indicating a medium level of adaptability. Several factors may explain this finding. First, the communication modes and styles of new nurses, colleagues, and leaders at work differed from those of new nurses, classmates, and teachers prior to employment. Second, the lack of work and life experience among new nurses may result in diminished confidence in managing interpersonal issues and heightened pressure when facing evaluations and instructions from leaders.^[31]^ Meanwhile, their limited professional skills and new clinical knowledge can hinder their ability to meet the patient’s needs, further impacting their workplace adaptability. Nursing managers should provide more support and attention to newly graduated nurses, expand the scope and depth of their training, and enhance interpersonal skills. Adopting formative evaluation^[32]^ and constructivist teaching^[33]^ methods can effectively promote their workplace adaptability.

The results of this study showed that the work readiness score of newly graduated nurses was 272.11 ± 45.70, which was slightly higher than the medium-level value reported by Gao et al.^[34]^ This observation may be attributed to several factors. By contrast, the low scores in work competence (clinical theory and skills, confidence, experience, and responsibility) and personal work characteristics (flexibility, stress management, and psychological resilience) indicated a certain gap in clinical requirements in theoretical knowledge and skills, confidence, stress management, and psychological quality. In clinical practice, new nurses encounter complex work environments, demanding training tasks, and stringent assessment requirements, all of which contribute to a reduction in their work readiness levels. However, the dimension of organizational acumen (maturity, professional planning, hospital policies, and systems) received the highest score, suggesting that new nurses possessed a relatively comprehensive understanding of the nursing work process and career planning. This contributed to their medium-level work readiness. Nursing managers should focus on enhancing new nurses’ work competencies and personal work characteristics to further improve their work readiness levels.

The study found that the mean score of each dimension of TS was 2.96 ± 0.72, indicating a medium level of shock. This may be attributed to the gap between theoretical and operational skills training received in colleges and universities and the values developed by new nurses in their clinical practice. With the rapid advancement of clinical technologies, the emergence of businesses, and the continual evolution of nursing concepts, swiftly mastering these elements quickly can be challenging. This often leads to confusion, doubt, and psychological stress.^[35]^

A significant positive correlation was observed between work readiness and workplace adaptability scores, indicating that higher work readiness levels are associated with better workplace adaptability among newly graduated nurses. Those with high work readiness demonstrate greater work performance and career development potential among newly graduated nurses. Newly graduated nurses with high work readiness were more likely to experience successful role transitions, higher nursing work satisfaction, and greater competence and independence in nursing duties, thereby enhancing their workplace adaptability.^[16]^

A significant negative correlation was found between TS and workplace adaptability, underscoring the importance of TS in improving workplace adaptability among newly graduated nurses. According to the TS theory,^[11]^ newly graduated nurses undergoing professional role transition encounter challenges in 4 key aspects: role, responsibility, knowledge, and interpersonal relationships. These challenges can heighten their difficulty in adapting to clinical work, negatively affect their perception of the work environment, and induce fear and avoidance of leadership, thus diminishing their workplace adaptability.

A significant negative correlation was found between TS and work readiness, indicating that lower TS levels were associated with better work readiness among newly graduated nurses. In the initial phase of role transformation, newly graduated nurses often experience substantial impact in all aspects due to unfamiliar clinical work and interpersonal relationships, a vague understanding of their role and professional significance, and high expectations from others. This can inevitably reduce their work enthusiasm and commitment, potentially leading to job burnout and difficulties in role adaptation,^[36]^ thus affecting their work readiness. These results suggest that nursing managers should strengthen collaboration between educational institutions and medical facilities, establish a robust cooperative framework, ensure adequate clinical practice opportunities, and provide effective training and support to improve newly graduated nurses’ work readiness.^[16]^ Additionally, offering professional knowledge and skills aligned with clinical practice standards along with psychological counseling interventions such as positive psychological group counseling, structural empowerment, and organizational socialization training can help minimize the negative impact of TS among newly graduated nurses.

The results of the structural equation model showed that TS partially mediated the relationship between work readiness and workplace adaptability, accounting for 44.01% of the total effect. Newly graduated nurses with higher work readiness demonstrated more independence in their roles and a stronger commitment to self-improvement, which enhanced their self-efficacy and job satisfaction, reduced their anxiety levels,^[37]^ and encouraged proactive responses to transition challenges. These positive outcomes further facilitated their workplace adaptability. Newly graduated nurses with lower work readiness scores may lack clear career planning; be unfamiliar with hospital culture, rules, and regulations; and exhibit poor work performance, all of which can impede their role transformation. The degree of TS had a significant impact on workplace adaptability. Therefore, nursing managers should prioritize addressing work readiness and TS among newly graduated nurses, fully understand the effects of role transformation, and proactively implement effective interventions (e.g., enhanced mentorship programs, peer mentoring, and resilience training workshops) to support these nurses. These measures would further enhance workplace adaptability, stabilize the nursing team, and support sustainable development.

The findings suggest several recommendations for future studies. First, the sample size was small, and this study focused on exploring only 2 tertiary hospitals in Wuhan, China. Conducting multicenter studies with a larger sample size would help validate the model and enhance the generalizability of the results. Future research should include more diverse samples from various regions in China. Second, this study did not explore the longitudinal or in-depth mechanisms underlying the influence of TS and work readiness on workplace adaptability. Further research should employ a longitudinal design and investigate additional variables to better understand the complex relationships and mechanisms involved. Long-term follow-up studies and qualitative approaches (e.g., interviews to explore TS experiences) could provide valuable insights into the dynamic nature of workplace adaptability in this population. Third, as the present investigation was conducted solely in China, caution should be exercised when extrapolating the findings to other countries due to the potential cultural differences between China and Western countries. Consequently, it would be beneficial to corroborate the outcomes of this study in newly graduated nurse cohorts from diverse nations while also acknowledging cultural heterogeneity in forthcoming research efforts.

## 5. Conclusion

Work readiness plays a significant role in regulating newly graduated nurses’ workplace adaptability. The study demonstrated that workplace adaptability was directly or indirectly affected by work readiness; in particular, TS significantly reduced the workplace adaptability of newly graduated nurses. The identified mediation effect accounted for 44.01% of the total effect. The substantial magnitude of this proportion underscored the pivotal role of TS. Given the novelty of the specific model, a lack of directly comparable literature was noted (e.g., Wang et al, 2022, had investigated a distinct multiple mediation model). Therefore, the value of 44.01% serves as a crucial benchmark for future research. We encourage scholars to test this specific model in different contexts to determine whether the potency of this mediation pathway is consistent across cultures and healthcare systems.

To improve the workplace adaptability status of newly graduated nurses, nursing managers should reduce TS and improve work readiness among newly graduated nurses through systematic intervention strategies (such as stimulating internal motivation, creating harmonious medical relationships, rationally arranging human resources, reducing work pressure, and improving the efficiency of nursing team cooperation and humanistic care^[38]^) to compensate for the limitations of the depth of workplace adaptability employed in further research.

## Acknowledgments

We would like to thank all newly graduated nurses who participated in this study.

## Author contributions

**Conceptualization:** Xiang Yi, Guoqing Wang.

**Data curation:** Xiang Yi, Guoqing Wang.

**Formal analysis:** Xiang Yi, Guoqing Wang.

**Investigation:** Xiang Yi, Guoqing Wang.

**Methodology:** Xiang Yi, Guoqing Wang.

**Supervision:** Xiang Yi, Guoqing Wang.

**Writing – original draft:** Xiang Yi, Guoqing Wang.

**Writing – review & editing:** Guoqing Wang.
